# Exploring the concept of old age in the Italian population: representations and cultural symbolism across different regions

**DOI:** 10.3389/fpsyg.2025.1583208

**Published:** 2025-06-02

**Authors:** Michela Di Trani, Barbara Cordella, Francesca Greco, Elena Argenti, Ambra Galiccia, Maria Gattuso, Andrea Greco, Francesca Morganti

**Affiliations:** ^1^Department of Dynamic, Clinical Psychology and Health, Faculty of Medicine and Psychology, Sapienza University of Rome, Rome, Italy; ^2^Department of Languages and Literatures, Communication, Education and Society, University of Udine, Udine, Italy; ^3^Department of Human and Social Sciences, University of Bergamo, Bergamo, Italy

**Keywords:** old age, culture, general population, interview, health

## Abstract

Global demographic trends show increasing life expectancy, with European countries such as Italy showing a high proportion of older adults. To support healthy aging and design effective health promotion interventions, it is crucial to understand how aging is defined and perceived. This study analyzes representations of aging among individuals in two different Italian cities to determine whether geographical context influences their perspectives. A total of 97 participants aged 55–75 years were interviewed about their experiences of aging, and the interview texts (145,066 tokens) were analyzed using Emotional Text Mining. Aging is perceived as a transitional process involving family responsibilities, changing work roles, and the retirement experience. Common concerns include mental and physical decline, often expressed through comparisons with participants’ past selves or with loved ones. Attitudes toward aging varied: some participants expressed resignation, while others adopted a more proactive approach, often centered around family or social engagement. Geographical and age-based differences were found: respondents from Bergamo were generally more accepting of aging, more focused on health concerns, and more socially active, whereas those from Rome placed greater emphasis on family care. Participants under 65 years of age primarily focused on the transition out of the workforce, while those over 65 years of age focused on the vulnerabilities associated with aging. These findings suggest that tailoring health promotion interventions to individual needs and contextual variables could enhance their effectiveness.

## Introduction

In 2015, the World Health Organization (WHO, 2015) released its first Global Report on Ageing and Health. In 2016, the Organisation for Economic Cooperation and Development (OECD) identified Italy as the longest-lived nation in Europe, second only to Japan worldwide. In 2022, the Italian National Institute of Statistics (Istat, 2022 – Istituto nazionale di statistica) outlined a demographic profile that reflects an aging trend: 12.7% of the population is aged 14 years or younger, 63.5% falls within the ages of 15 and 64, and 23.8% is aged 65 years or older. Projections indicate that by 2050, individuals over 65 years of age could account for 34.5% of Italy’s total population.

With respect to the concept of aging, a systematic review by López-López and Sánchez (2020) highlighted its evolution, from a perspective primarily focused on pathology and dementia to one that emphasizes the promotion of wellbeing and the need for a multidimensional approach. For example, studies such as that by Rodrigues et al. (2019) propose a link between cognitive functions and physical performance, suggesting that a decline in one may serve as a predictor for a decline in the other (Basile and Sardella, 2021). With this background, the strict welfare-based vision was overcome; older adults are not considered passive citizens in need of assistance but rather adopt a proactive role that consciously embraces both the limitations and resources of aging (Martineau and Plard, 2018; Morganti, 2022; Ebner et al., 2023). Therefore, we do not focus only on pathological aging, associated with dementia and loss of autonomy (World Health Organization, 2020), but also consider a broader concept of aging as originally defined by the WHO (2002): “the process of optimizing opportunities for health, participation and security in order to enhance the quality of life as people age.” This condition is affected by several dimensions, including physical health, psychological state, level of autonomy, social relationships, personal beliefs, and the broader network of individual, group and community relations (Bertini, 2012), aligning with a systematic view of aging grounding in the understanding of health as “a state of complete physical, mental, and social well-being.”

Therefore, conceiving aging as not necessarily equivalent to a condition of disease and passivity allows us to broaden the scenario of complexity (Mustafina and Somova, 2015; Burch et al., 2014; Castillo-Chuan and Kinghorn, 2013; Friedman, 2020), but we may question the extent to which this perspective is shared by both the general population and by experts in the field.

Understanding how people represent aging becomes a source of information necessary to build promotional interventions, considering the context in which people live and their needs. Studying social representations (Moscovici, 1976, 2000) means collecting how common sense relates to the topic of aging, to values, beliefs, and social practices, which guide the thoughts and behavior of individuals. Studying social representations, in short, is a way to study the socially shared meanings on a topic in a given time (Salvatore and Cordella, 2022).

In research conducted in Italy in 2010 (Zanbianchi and Ricci Bitti), it emerged that the stage of old age possesses resources and the potential for personal growth, also, through the maintenance of a future, the valorization of wealth due to experience, and the possibility of finding positive ways to face and compensate for the decline in health. This is also confirmed by Contarello et al. (2011), who describe aging as a compromise between biological conditions and individual experiences, starting from the linguistic analysis of two Italian narratives on this topic.

Similar results are also found in Contarello and Romaioli (2020), who interviewed young people, adults, and older adults, noting that it is precisely older adults who express the most positive representation of aging. However, this depends on the economic and social conditions in which one lives and the presence of grandchildren and children. Nevertheless, according to the authors, this conception of old age is episodic, anecdotal, and does not yet present itself as a new social representation. Rather, it is narrated in contrast to the dominant view of old age as a period of decline and loss.

Following this perspective, it is interesting to consider the work of Mendes et al. (2012), who examined the social representations of aging developed by health professionals. The results indicated five categories: vision of aging, psychosocial dimensions, a time of doubts, aging as a process, and aging versus disease. Some positive aspects were linked to content such as joy, care, children, retirement, rights of those who take care of them, maturity, and wisdom. Other negative contents were linked to impairments, deterioration, abandonment, fragility, limitation, wrinkles, dependence, and disease. In some cases, professionals can have negative opinions about aging while trying to promote positive opinions in their work, illustrating a gap between theory and practice (Craciun and Flick, 2016). In other cases, job experience influences the representation of aging in health care workers (Swift and Steeden, 2020), highlighting the role of several variables in the social construction of representations.

Thus, if it does not appear possible yet to define a new, fully accomplished representation of aging, it has highlighted the need to review the representations of aging populations to implement a process of change and acceptance of diversity, promoting new representations of aging set in specific contexts (Ebner et al., 2023). Cultural representations are the premises for the implementation of healthy behaviors. They act on the preventive actions that individuals and the community can foresee. In this perspective, a study (Monzani et al., 2022) highlighted how thinking about the future as an older adult increases the perceived risk of disease and can activate protective behaviors. Therefore, acting on people’s representations as they approach old age can orient their future thinking; this could be a successful strategy to generate awareness and preventive actions.

In line with what has been said, current policies on aging support the need for “system actions” (Barbabella et al., 2022), moving away from the perspective of understanding and intervening on the individual and rather exploring and acting on the cultural and social context.

### The present research

According to the literature, a revision of the dominant representations of old age is underway, although these new representations have not yet fully consolidated. While research on aging is increasingly moving away from universal models focused on biological changes over the life course (see, e.g., Rowe and Kahn, 2015), there still appears to be limited attention to the contextual and relational factors that shape the interaction between individuals and their environments. These factors are essential for restoring a subjective dimension to the understanding of successful aging. In this regard, analyzing local cultural representations of aging may offer valuable insights into the individual and contextual dynamics involved in the aging process and may help identify specific areas for intervention that are closely tied to local and territorial contexts.

For this reason, we aimed to explore how representations of aging are structured in two distinct areas of the country: Bergamo and Rome. These cities differ significantly in terms of geographical location (northern vs. central Italy) and population size, making them suitable for comparison. Based on our initial hypothesis, these contextual differences may influence how aging is represented. To deepen the analysis, we investigated differences based on age, working status, and gender. Participants between the ages of 55 and 75 were selected to examine whether representations vary between those still in the workforce and those already retired, and to identify any gender-based distinctions.

In short, this study explores the following questions:

Q1: Which representations of old age emerge?

Q2: If these representations differ according to the territory.

Q3: Do these representations differ according to the variables of age, gender, and employment status?

## Methods

### Procedure and participants

The study was planned and carried out by the Department of Dynamic and Clinical Psychology and Health Studies at Sapienza University of Rome and the Department of Humanities and Social Sciences at the University of Bergamo. This is part of a PRIN research project, “Development and testing of psycho-promotion indicators for a successful aging of older adults,” funded in 2022. This study was approved by the Ethics Committee of the University of Bergamo, Italy, in accordance with the ethical principles established in the Declaration of Helsinki and in the Convention on Human Rights and Biomedicine (Oviedo Convention).

A group of 97 participants was selected. We predefined two inclusion criteria:

- Age: 55 to 75 years old
- Geographical area: A major city in northern Italy and one in central Italy.

Those who could not take the interview for language reasons or health conditions were excluded. Respondents were recruited with a snowball method. Rome (central Italy) and Bergamo (northern Italy) were the cities. Participants were contacted by telephone, and if they agreed to the interview, a researcher conducted it in their home. They were asked to complete an informed consent form prior to the interview.

### Data collection

We used an open-ended question interview to support research participants’ narration of their experience of aging (Flick, 1998). The question was the following:

“As researchers, we are trying to understand how people cope with old age: the resources they can draw on, the difficulties they face, and how they relate to this transition. So, we are very interested in hearing about your experience.”

After posing the stimulus question, the interviewer utilized additional prompting (i.e., ‘echo responses’) to facilitate the exploration of participants’ experiences of aging. This prompting was employed only when the interviewee’s silence indicated a premature end to their response, ensuring that the interviewer did not interrupt the interviewee’s train of thought. The interviews lasted 30 min on average. At the end of the interview, personal data were collected (age, sex, geographical area, and working status).

### Data analysis

Audio-recorded interviews were transcribed verbatim and collected in a corpus that was analyzed by means of a natural language processing procedure: Emotional Text Mining (ETM – Greco, 2016a, 2016b; Greco and Polli, 2020) to investigate the representation and cultural-symbolic categories of aging that emerge from the interviews. Transcripts were labelled with two illustrative variables: geographical area (Rome, Bergamo) and age by working status (55–64 years old, 65–75 years old). ETM is a bottom-up, unsupervised procedure based on a socio-constructivist approach and a psychodynamic model that was largely used in social sciences (e.g., Greco et al., 2020; Greco and Polli, 2021; Boccia Artieri et al., 2021; Fronzetti Colladon et al., 2023) and particularly in psychology (e.g., Cordella et al., 2011, 2023; Di Trani et al., 2021).

To determine the corpus’s suitability for statistical analysis, two lexical indicators were calculated: the type-token ratio (TTR) and the hapax percentage (hapax%). The data were then cleaned and preprocessed, and the corpus was segmented into comparable text chunks. Terms of medium and low frequency were selected, up to a threshold equal to the total number of modalities of illustrative variables (Greco, 2016a), to perform the multivariate analysis using lemmas rather than types. Stop words were filtered out.

We conducted a cluster analysis using the bisecting k-means algorithm based on cosine similarity (Steinbach et al., 2000), applied to the term-per-chunk-of-text matrix. The analysis was limited to 20 partitions and excluded all text chunks that did not contain at least two co-occurring terms to identify aging representations (Greco and Polli, 2021). We evaluated three indices to determine the optimal number of clusters: the Calinski-Harabasz index, the Davies-Bouldin index, and the intraclass correlation coefficient. Subsequently, a correspondence analysis (Lebart and Salem, 1994) was performed on the cluster-by-term matrix to identify symbolic categories (Greco and Polli, 2020). To interpret the results of the ETM, three independent judges—each trained in the method—reviewed the data separately to identify participants’ symbolic categories and aging representations. Their interpretations were then compared, and the most coherent labeling, i.e., the one best fitting the data, was selected. Finally, we performed chi-squared tests to assess the distribution of the clusters across the illustrative variables (age, geographical area, gender, and working status) (Capogna and Greco, 2024; Donald, 2015). To evaluate the representativeness of each text chunk, we calculated a score based on the weighted sum of the chi-squared values of the terms occurring in that chunk.

To identify the symbolic categories of aging, three independent judges, each with psychological and sociological training and qualification in EMT, independently interpreted the results of both the correspondence and the cluster analyses. This interpretative methodology involves reading all the terms, assigning each a symbolic meaning, and attributing an appropriate label. Subsequently, the process includes comparing the three interpretations and, if necessary, integrating them to select the labels that most accurately reflect and represent the data.

## Results

A total of 97 participants, aged between 55 and 75 years old, were voluntarily recruited from two Italian cities (Bergamo and Rome) and interviewed about their experiences of aging. Among the residents of Bergamo (*N* = 50), 76% were female, and the median age was 63 years (IQR = 10.75), with 46% being older adults. The majority reported living with others (76%) and being actively employed (64%). Among the residents of Rome (*N* = 47), 55.32% were female, and the median age was 65 years (IQR = 9.5). 51.06% were older adults. Similarly, most of them reported living with others, but unlike the Bergamo sample, more than half of the Roman participants (55.32%) reported not being active in the workforce. Specifically, 19 were retirees, 2 were unemployed, and 5 women reported being homemakers.

The transcription interviews from 97 participants generated a large corpus (145,066 tokens). It was segmented into 2,017 comparable text chunks. The lexical indices suggested the feasibility of performing ETM (TTR = 0.072; hapax = 49.9%). The results of the cluster analysis indicated that the 811 selected terms classified 99.7% of the text chunks. The optimal partition resulted in six thematic clusters. Correspondence analysis identified five factors setting the symbolic space in which clusters are located, defining the emotional framework associated with aging. The explained inertia for each factor is reported in Table 1.

**Table 1:** Correspondence analysis results.

Factor	Eigenvalues	Percentage	Cumulative percentage
1	0.1674	30.0	30.0
2	0.1060	19.0	49.0
3	0.1037	18.6	67.6
4	0.0980	17.6	85.2
5	0.0824	14.8	100.0

The first three factors explain 68% of inertia, and their interpretation is reported in Table 2.

**Table 2:** Correspondence analysis interpretation (the first 10 terms of each pole are listed in descending order based on their absolute contribution percentage).

Factor 1 - Trajectory		Factor 2 - Commitment		Factor 3 - Limit	
Negative pole	Positive pole	Negative pole	Positive pole	Negative pole	Positive pole
Dynamic	Static	Family	Social	Resignation	Reactive commitment
Change	House	Son	Dad	Think	Work
Face	Understand	Family	Us	Old age	You
Different	Problem	PUT	Succeed	Scare	Elderly
Health	Husband	Grandmother	Group	Search	Example
Believe	Loneliness	Question	Initiatives	Difficulty	People
Respect	Mother	Possible	Help	You	Prevention
Accept	Resource	Kids	Social	Worry	University
Better	Grandchildren	True	Hard work	Watch	Happens
Rest	Mother	Theater	Find	Luck	Menopause
Situation	Evening	Cinema	Attitude	Mind	Reality
1.23–0.37 a.c.%	4.08–1.42 a.c.%	4.79–0.15 a.c.%	3.99–0.40 a.c.%	2.47–0.27 a.c.%	12.03–0.57 a.c.%

a.c.%, absolute contribution.

The words that characterize the first factor appear to indicate two opposite ways of prefiguring old age. The negative pole refers to change, the need to accept a period different from the one previously experienced, and highlights the proactivity required to face the anticipated challenges of the situation. On the other hand, the positive pole reflects a sense of closure, the start of a period characterized by problems and solitude, confined within the home, both as a physical space and as a social environment, aimed solely at the closest relationships. In short, we can refer to this factor using the term ‘trajectory’, distinguishing a dynamic position (negative pole) from a static one (positive pole). The second factor appears to argue for the attention paid to the internal world of the home versus what is external to it. Thus, the negative pole proposes terms such as child, family, grandparents, and boys, as opposed to the positive pole, in which the social dimensions emphasize the possibility of being a group, starting initiatives, or perhaps retiring. The two poles can represent different ways to face the experience of aging; we think it is possible to name this factor as ‘commitment,’ aimed in the two directions indicated, family vs. social. Finally, the third factor captures the dimension of the ‘limit,’ a term that we propose to mean the axis. For an older adult, looking ahead might be scary and induce worry. One can confront these thoughts either through logic aimed at resignation (negative pole) or through the search for a reactive commitment with respect to one’s own experience (positive pole).

In the previously indicated space of meaning (see Table 3), we consider clusters as the themes that are addressed by the interviewees (see Table 4).

**Table 3:** The position of the clusters with respect to the factors.

Cluster	CT%	Factor 1	Factor 2	Factor 3
1	16.07%	Dynamic	Social	Resignation
		−0.3439	0.2872	−0.1858
2	18.14%	Static	Social	Reactive Commitment
		0.4839	0.5636	0.3948
3	12.24%	Dynamic	Family	Reactive Commitment
		−0.3947	−0.5120	0.1971
4	24,36	Static	Family	Resignation
		0.5626	−0.3285	−0.3555
5	13,08	Dynamic	Social	Resignation
		−0.5467	0.3471	−0.4491
6	16,11	Dynamic	Family	Reactive Commitment
		−0.2376	−0.2971	0.5165

The table shows the placement of clusters with respect to the three factors. The second column reports the percentage of the chunk of text (CT) included in the individual cluster.

**Table 4:** The words that characterize the clusters.

Cluster 1		Cluster 2		Cluster 3		Cluster 4		Cluster 5		Cluster 6	
Conscious transition		Between past and present		Beyond work		Family		Vulnerability of the body		Keeping busy	
Term	CT in CL	Term	CT in CL	Term	CT in CL	Term	CT in CL	Term	CT in CL	Term	CT in CL
People	142	Example	128	Life	328	Son	210	Think	136	Work	283
Feel	124	Us	121	Work	81	House	143	Physical	114	You	126
Age	120	Elderly	120	Live	46	Problem	132	Grow old	114	Retire	108
Live	103	Succeed	69	You	46	Understand	120	Old age	64	Put	46
First	101	Father	67	Change	38	She	97	Sure	53	Change	44
Watch	100	Help	61	Family	27	Loneliness	93	Believe	35	Activity	37
Difficulty	57	Need	57	Became	20	Resource	86	Health	33	Different	25
Old	55	Mother	45	Face	20	Husband	67	Mental	32	Continue	20
Young	54	Find	36	Reality	16	You	64	Face	32	Difficult	20
Rest	43	Mother	34	Rest	16	Family	61	Aspect	25	Reality	20
Repeat	42	Our	30	Future	16	Grandchildren	53	Level	24	People	19
Arrive	39	Group	27	Theater	11	Manner	45	Scares	20	Kids	16
Faith	34	Initiatives	20	Passage	11	Money	40	Body	20	Finished	11
Question	15	Read	18	Better	9	Evening	37	Scare	17	Euro	10
Awareness	12	Prevention	13	Ugly	7	Tonight	36	Nutrition	10	Contact	10

The table shows the first 15 words that characterize the single cluster (CL) and the number of chunks of text (CT) of each word. So, CT in CL: number of chunks of text present (chunk in which the term is present) in the cluster.

Cluster 1, named ‘Conscious Transition,’ appears to propose the oscillation of those who still feel young but, simultaneously, also old between the desire to live and the difficulties related to advancing age, thus building their awareness of the transition they are facing (Table 5).

**Table 5:** Some of the CTs associated with cluster 1, “Conscious Transition,” and participants’ characteristics.

Text-ID 52–55-65 age group – Rome – Working – Female – CT score: 484,1,565
The difficulties clearly come with growing older. You take a few steps and start sweating, whereas before, you used to run marathons – now you take a few steps and sweat. You have less patience with other people, so you feel less willing to waste time with those who, in your opinion, are not worth it. Unlike before, when you opened up to whoever was around without caring, it’s not the same now.
Text-ID 78–66-75 age group – Rome – Not working – Male – CT score: 481,7,027
Here it is - these are the things that get in your way and constantly remind you that you are growing old. And then you have people saying, “Oh, but you know, that thing is important.” You do not believe it - it’s hypocrisy. “What matters is staying young at heart,” they say, but not even that feels true. That idea, let us say, only makes it worse in a certain sense.

Cluster 2, named ‘Between past and present,’ begins with the word ‘example,’ a significant term both if it is considered in terms of the example we are looking at (the seniority seen in previous generations) and if it is considered as what can still be transmitted. Father, help, need, and mother are terms that appear to signal a look to the past, to the care of grandparents and parents. The word ‘find’ follows, that is, looking for a new way that relies on the group, initiatives, prevention, and the need itself. In short, to find a place as an older person in a world different from the previous one (Table 6).

**Table 6:** Some of the CTs associated with cluster 2, “Between past and present,” and participants’ characteristics.

Text-ID 79–66-75 age group – Rome – Not working – Male – CT score: 1270,9,571
Instead of, I say, going to play, let us make a city for the elderly where they take their children and there is a nursery run by elderly people and, there is, the supermarket run by elderly people; that is, if society organized in my opinion structures run completely from A to Z by elderly people, the elderly still feel alive instead of feeling dead at home. That’s all.
Text-ID 17–55-65 age group – Bergamo –Working – Female – CT score: 873,2,316
In my work, I often see elderly people. I have seen so many elderly individuals who have cared for my parents in the past, and even back then, the enormous gap was evident. Some had someone close to them, someone doing something for them, and others had no one at all. It was, and still is, truly a devastating sight.

In cluster 3, named ‘Beyond work’, the first two words, life and work, appear to indicate the activity that occupies one’s time. It is followed by ‘living’, which appears to indicate both the continuation of life beyond work and the hope of participating in life’s activities at a different time, outside of work commitments. Then ‘you,’ the search for the other and ‘change,’ are followed by becoming, facing reality, the rest of life, and the future—it will be a better or worse passage (Table 7).

**Table 7:** Some of the CTs associated with cluster 3, “Beyond work,” and participants’ characteristics.

Text-ID 96–66-75 age group – Rome – Not working – Male – CT score: 613,6,628
The point is that I consider myself lucky because I am sixty-eight years old and seem to be in relatively good health. In contrast, my father passed away at forty, my mother at sixty-eight, and my sister at sixty-four. For me, aging is a positive thing because it’s better to tell the story than to die young, so that’s my perspective.
Text-ID 13–55-65 age group – Bergamo – Working – Female CT score: 194,4,028
Peaceful - that’s how I feel. However, when I think about myself and my old age, it scares me, and I do not feel as optimistic. I hope to be pleasantly surprised in the future and gradually see that I, too, will manage to remain active, hopeful, and have that spirit I notice in many people of a certain age who seem to find their way.

Cluster 4, named ‘Family’, begins with the word ‘son’, which recalls the theme of continuity and the resource on which one can rely, the ‘family’ as a reference. The word ‘home’ follows, which in this context appears to recall a safe place, followed by ‘problem’ and ‘understand’. Moreover, this cluster highlights how the reference to known models is reassuring and uncertain. Husband, family, and grandchildren could be a resource against loneliness, a possibility of thinking of oneself as a group, to stay together when one imagines, for example, the arrival of the evening, but without denying the uncertainty in which all of this is placed (Table 8).

**Table 8:** Some of the CTs associated with cluster 4 “Family” and participants’ characteristics.

Text-ID 95–66-75 age group – Rome – Working – Female – CT score: 321,9,488
Family may come to mind but at this age family is relative… eh because at this age the children, those who have children, eh are grown up. The grandchildren more or less even if they are small still have parents, let us say a sporadic activity that can be entrusted to the grandparents and then, which grandchildren? Who has grandchildren anymore?
Text-ID 43–66-75 age group – Bergamo – Working – Male – CT score: 311,261
It also depends on whether you end up alone, if the wife is left alone, or if the husband passes away first. There are many factors, I think, at least this is my interpretation of the question… The resources, of course, refer to financial resources. If you have some resources, it’s possible that someone might come closer to help. If you do not have resources, as often happens in Italy, no one approaches at all.

The words that appear in cluster 5 refer to health (both mental and physical), the aging process that leads one to old age, and a loss of one’s performance, a resource that is limited and must be addressed based on what is lacking and the degree of impairment. This is how the interviewees envision the future (Table 9).

**Table 9:** Some of the CTs are associated with cluster 5, “Vulnerability of the body,” and participants’ characteristics.

Text-ID 34–66-75 age group – Bergamo – Working – Male – CT score: 393,2,568
What I imagine about aging, I’m obviously talking about later, I imagine that the difficulties that one must encounter are precisely those of physical decay, of physical aging…
Text-ID 14–55-65 age group – Bergamo – Working – Female – CT score: 364,9,332
Physical changes—well, I’d say I’m somewhat of a fatalist in the sense that I accept what comes as it comes. At a certain age, you realize that your body changes. Menopause has already brought about radical changes, and you have to deal with issues like weight gain as well as the problem of overexertion.

Finally, cluster 6, named ‘Keeping busy,’ starts with the word ‘work’ followed by ‘pension.’ The words that follow describe a difficult transition: continuing to work or finding something else to occupy the time, keeping in touch with others, being with people, and staying active (Table 10).

**Table 10:** Some of the CTs associated with cluster 6, “Keeping busy,” and participants’ characteristics.

Text-ID 35–66-75 age group – Bergamo – Working – Female – CT score: 248,73
Since I’ve retired and am staying at home, I’m not the type of person who enjoys sitting around watching television or wasting time. Once I’ve taken care of my household tasks, that’s it—I’m done. I go out or find something to do because I do not sit in front of the television. Instead, I read, and since retiring, I’ve been reading quite a lot of books.
Text-ID 55–55-65 age group – Rome – Working – Male – CT score: 231,2,934
If you look at my phone, I download all sorts of things—home workout routines, things I can do, things I know how to do, and more. But they all just stay there. I do not lie in bed on Saturdays and Sundays doing nothing. I watch movies during the night because I do not sleep much. Perhaps it’s a deeper satisfaction that I still cannot seem to fill.

Overall, there was a statistically significant difference in participants’ perceptions of aging based on geographical area and age group (χ^2^, df = 5; *p* < 0.05), as highlighted by the standardized residuals reported in Table 11. In contrast, no statistical association was found between working status or gender and aging representation (clusters) (χ^2^, df = 5; *p* > 0.05). The representations of ‘conscious transition’, ‘between past and present’, ‘beyond work’, ‘family’, and ‘vulnerability of the body’ (clusters 1 to 5) differ by geographical area, while the ‘keeping busy’, and ‘vulnerability of the body’ representations (clusters 5 and 6) differ by age group.

**Table 11:** Participants’ specificity related to the geographical area and age group (the standardized residual is reported in each cell).

	Age		Geographical area	
	55–64	65–75	Bergamo	Roma
Conscious transition	1.1	−1.2	**5.2**	**−4.5**
Between past and present	0.0	0.0	**−4.1**	**3.5**
Beyond work	−1.3	1.5	**3.4**	**−3.0**
Family	0.2	−0.2	**−5.7**	**4.9**
Vulnerability of the body	−1.9	**2.1**	**3.0**	**−2.6**
Keeping busy	**2.2**	**−2.5**	−0.1	0.1

Specifically, only participants aged 55 to 64 years tend to adopt the ‘keeping busy’ representation, likely because they are more frequently still working. This representation is significantly less present among participants aged 65 to 75, who focus more on the ‘vulnerability of the body’. Interestingly, participants from northern Italy are characterized by representations of ‘conscious transition’, ‘beyond work’, and ‘vulnerability of the body’ (clusters 1, 3, and 5), while those from central Italy tend to focus more on the ‘between past and present’ and ‘family representations’ (clusters 2 and 4). It is worth noting that clusters 3 and 5 stem from cluster 1, while clusters 4 and 6 stem from cluster 2. This suggests that factor 1 likely holds strong cultural significance in shaping participants’ narratives.

## Discussion

This research has pursued a threefold purpose. This study explored the representations of the third age (Q1) in relation to the consideration of geographical contexts (Q2) and in relation to the age variables (55–64; 65–75), gender, and working conditions (Q3). With these objectives, the semantic field (meaning attributed to the factors) was read, and the location of the different clusters was considered. As indicated in Table 1, the first factor – the most significant science – explains the highest percentage of inertia. It distinguishes the semantic field into two distinct areas that correspond to the way in which one relates to old age (which trajectory is assumed), one static, the other dynamic. We speak of trajectory precisely because we imagine two different perspectives, one (dynamic) oriented *to accept* a new adaptation, aimed at discovering the *changes*^1^ necessary to face a *different situation* because of one’s *health* conditions, which may also include *rest*. The other (static), which moves in a more restricted field, that of the *house*, with its members (*husband, mother*), appears dominated by *solitude* and approaches the *evening*, counting on a single resource: *the grandchildren.*

Factors 2 and 3 are useful for making possible distinctions in the configuration proposed by factor 1. Thus, factor 2 speaks of the places where one spends one’s daily commitment: family (negative pole) and social (positive pole). It can be noted that among the words of the pole that define society, we find *ourselves*, *group*, *social*, and a context of belonging in which to offer and *find help* through *initiatives* and *hard work*. At the opposite pole (negative), next to the words that recall family roles (*son, grandmother, children*), we also find *cinema* and *theater*, two places that we can consider external and for leisure, but that make us think of people sitting by participating in the scene conducted by others.

Finally, factor 3 is defined as the factor that speaks of the “limit” that we encounter and how we relate to it. *Fear*, *difficulty*, *worry*, and *mind* are the words that characterize the negative polarity (resignation). For *example*, *prevention*, *university*, and *reality* are the words present in the positive polarity (reactive commitment). Here, what appears relevant to us is that, in the negative polarity, the iterative one encountered something unexpected (or until that moment denied), whereas in the positive polarity, the notion of a limit appears to have been considered, perhaps due to the example received and the possibility of prevention.

**Figure 1:**
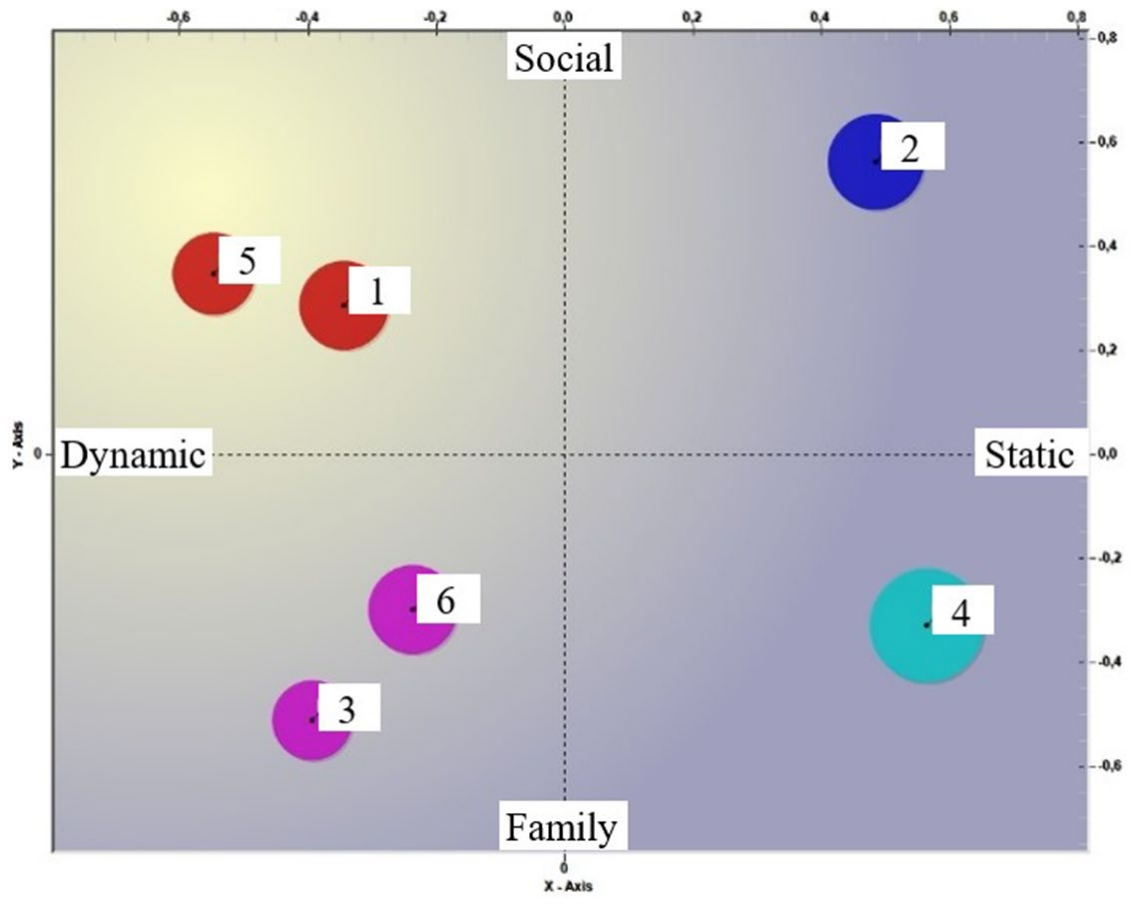
Clusters in factors 1 and 2.

**Figure 2:**
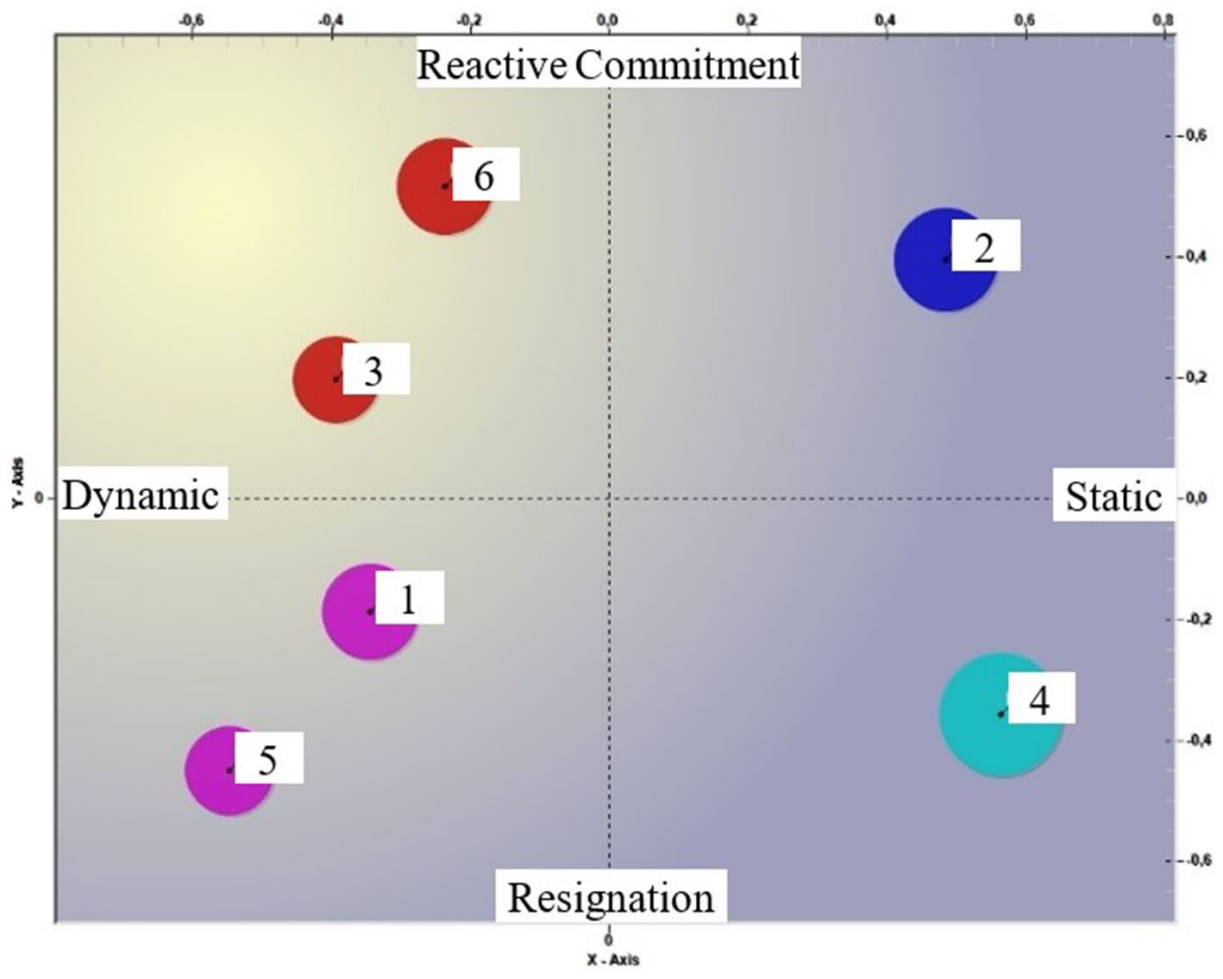
Clusters in factors 1 and 2. Figures 1, 2 show the location of clusters in the semantic field, specified with the meaning of the individual poles.

It is in this semantic field that the 6 clusters are located (see Figures 1, 2). Looking at Figure 1, it appears interesting to note that the clusters located in the Dynamic/Social quadrant (clusters 1 and 5) are positioned on the negative polarity of the third factor (Figure 2, Resignation). This finding appears to confirm the denial of the ‘limit,’ as previously discussed, but it can be read as an insufficiency of the anchoring to the social when abilities are lost. We also find cluster 4 on the resignation pole (Figure 2), located, as far as the first two factors are concerned, in the Static/Family quadrant (Figure 1). In this case, the cluster’s position within the resignation domain suggests a different interpretation: cluster 4 tells of the uncertainty the anchoring to the family can face. As a common factor, therefore, we could say that fear and worry arise from noting the insufficiency of the context to which we have referred. Illness, and even more so the loss of lucidity, calls into question the social relationships that until that moment have supported the older person.

On the “reactive commitment” pole of factor 3 (Figure 2), instead, are clusters 2, 3, and 6. Cluster 2, which, compared to the first two factors, is placed in the static/social quadrant, while Clusters 3 and 6 are placed in the dynamic/family quadrant. If cluster 2 recalls the learning received in the family regarding the life cycle, clusters 3 and 6 appear to strongly underline the need to prepare for a different time.

As regards the variables, as reported in Table 11, the subjects of Bergamo are represented by clusters 1, 3, and 5, while they are significantly not represented (presence of the minus sign) by clusters 2 and 4.

On the other hand, the interviewees in the city of Rome are characterized by clusters 2 and 4, while they are significantly not represented by clusters 1, 3, and 5. It appears, therefore, that the idea that representation is linked to the life context of the interviewees can be confirmed. It can be observed that clusters 2 and 4 are positioned on the positive pole of the first factor, to which we have attributed the meaning of static trajectory, while clusters 1, 3 and 5 are positioned on the negative pole of the first axis, to which we have attributed the meaning of dynamic trajectory. In summary, the two polarities express the representations emerging in the two cities. The subjects in Rome report the theme of family, of caring for their loved ones, expressing the desire to be with their children and grandchildren, while also recognizing the difficulty that this entails and the need to find meaning in their age.

The interviewees in Bergamo, by contrast, emphasize that they still feel more oriented towards seeking commitments external to the family that keep them active and show greater concern for a possible physical and mental decline. It appears that for the Rome interviewees, family is considered a valuable and important resource in approaching aging, which is imbued with this dimension of care and nurturing given and received over time.

For the Bergamo respondents, however, the primary sources appear to be related to dynamic, interpersonal, and relational dimensions such as maintaining physical and mental well-being and staying active and engaged to cope with aging effectively. Finally, regarding age groups, while respondents aged between 65 and 75 are associated with cluster 5, respondents aged between 55 and 65 are associated with cluster 6. This is not surprising if we consider that cluster 5 talks about the difficulty of dealing with a possible physical and mental decline, while cluster 6 talks about the end of work and the need to spend one’s time in another way. No differences in the representation of aging appear to emerge based on the sex of the subjects compared to the variables that do influence, such as age, geographical location, and culture. It is indeed possible that, like what Carstensen posits in the socioemotional selectivity theory (1987), where temporal dimension is a determining factor in guiding individuals’ motivations and behaviors throughout their life span, the adult interviewees who are closer to retirement might consider it an important issue to address and manage in the representation of aging. In fact, retirement is not only an individual event, but it can also be considered a social transition, often determining the recognition of an older person as such because they are no longer part of a productive economy. Conversely, interviewees who have already experienced retirement might be more focused on the fear of physical or cognitive decline, which, once again, is socially attributed to the older population and associated with advancing age.

### Implications for practice

We dedicate this paragraph to some considerations relating to the interventions that arise from the results of our research and lead to the design of different interventions in different territories. Based on these findings, the representations appear to outline scenarios in which it is possible to identify implicit needs, read requests for intervention, or, in other cases, take them into account to implement health promotion interventions. Similar results are also found in Contarello and Romaioli (2020), who interviewed young people, adults, and older adults, noting that it is older adults who express the most positive representation of aging, even if the latter depends on the economic and social conditions in which one lives and, in particular, on the presence of grandchildren and children.

Following this rationale, for example, a health promotion intervention in the Roman territory could prioritize focusing on caregivers, a role that older adults attribute to themselves and expect to be fulfilled within the family in the future (World Health Organization, 2020). In fact, support outside the family does not appear to be contemplated. Therefore, if the theme of the inevitable arrival of frailty allows us to imagine a sufficiently obvious place of care, it is also true that individuals tend to represent themselves within a perspective that only marginally allows for the anticipation of caregiving activities beyond the family sphere (Salvatore and Cordella, 2022). The underlying need for this representation appears to be that of support and accompaniment of families in the caregiving role attributed to them. Additionally, a hypothetical intervention could focus on shifting away from the static and closed dimension shared by the Roman population in the study towards aging. Such efforts could allow for not only the construction of new resources and the reduction of certain risk factors associated with aging (e.g., loneliness and depression) but also alleviate the caregiving responsibilities placed on family members.

On the other hand, a hypothesis of intervention in the city of Bergamo could be the prefiguration of reliable places of care in conditions of fragility, for example, the solidarity condominiums that are being tested in some countries of the world to improve and promote the social dimension. Moreover, involving local entities, both institutional and non-institutional, in the city that are engaged in offering initiatives dedicated to old people in the planning of intervention hypotheses could be the key to addressing individuals’ needs through opportunities for social encounters or tailored activities.

In both situations, the intervention could act on contextual variables rather than looking only at individual needs (Martineau and Plard, 2018; Morganti, 2022; Ebner et al., 2023). Therefore, if it does not yet appear possible to define a new, fully complete representation of aging, the need to review the representations of older populations is highlighted in order to implement a process of change and acceptance of diversity, promoting new representations of aging inserted in specific contexts (Ebner et al., 2023). Cultural representations are the premises for the implementation of healthy behaviors. They act on preventive actions that individuals and the community can foresee.

Therefore, acting on people’s representations as they approach old age can orient their future thinking; this could be a successful strategy to generate awareness and preventive actions. In line with what has been said, current policies on aging support the need for “system actions” (Barbabella et al., 2022), moving away from the perspective of understanding and intervening on the individual to explore and act on the cultural and social context.

## Conclusion

According to the results, the research supports the hypothesis that the representations of aging are linked to the territorial context of belonging (Q2) and that different representations are identifiable (Q1), as confirmed by other studies (Mustafina and Somova, 2015; Burch et al., 2014; Castillo-Chuan and Kinghorn, 2013). Overall, there was a statistically significant difference in participants’ perceptions of aging based on age group, while variables such as gender and employment status were not significant (Q3). However, the data supporting our hypothesis are not statistically significant, as they are limited to only two geographical contexts (Rome and Bergamo). It is important to consider that our research is constrained by the use of a convenience sample, which limits the generalizability of the results. From a research perspective, it would be necessary to explore these representations in other contexts in the future to enable more meaningful comparisons. Although gender and employment status were not significant in this study, future research should aim to include balanced groups with respect to these variables. Additionally, it would be useful to examine representations of old age as expressed by caregivers and healthcare professionals.

## Data Availability

The original contributions presented in the study are included in the article/supplementary material; further inquiries can be directed to the corresponding author.

## Footnotes

^1^We mark in italics the words taken from the factors.
